# Supplementary figures and images for: Role of Exopolygalacturonase-Related Genes in Potato-Verticillium dahliae Interaction
Source: Pathogens. 2021 May 23;10(6):642. doi: 10.3390/pathogens10060642 (PMC8224799; doi:10.3390/pathogens10060642)

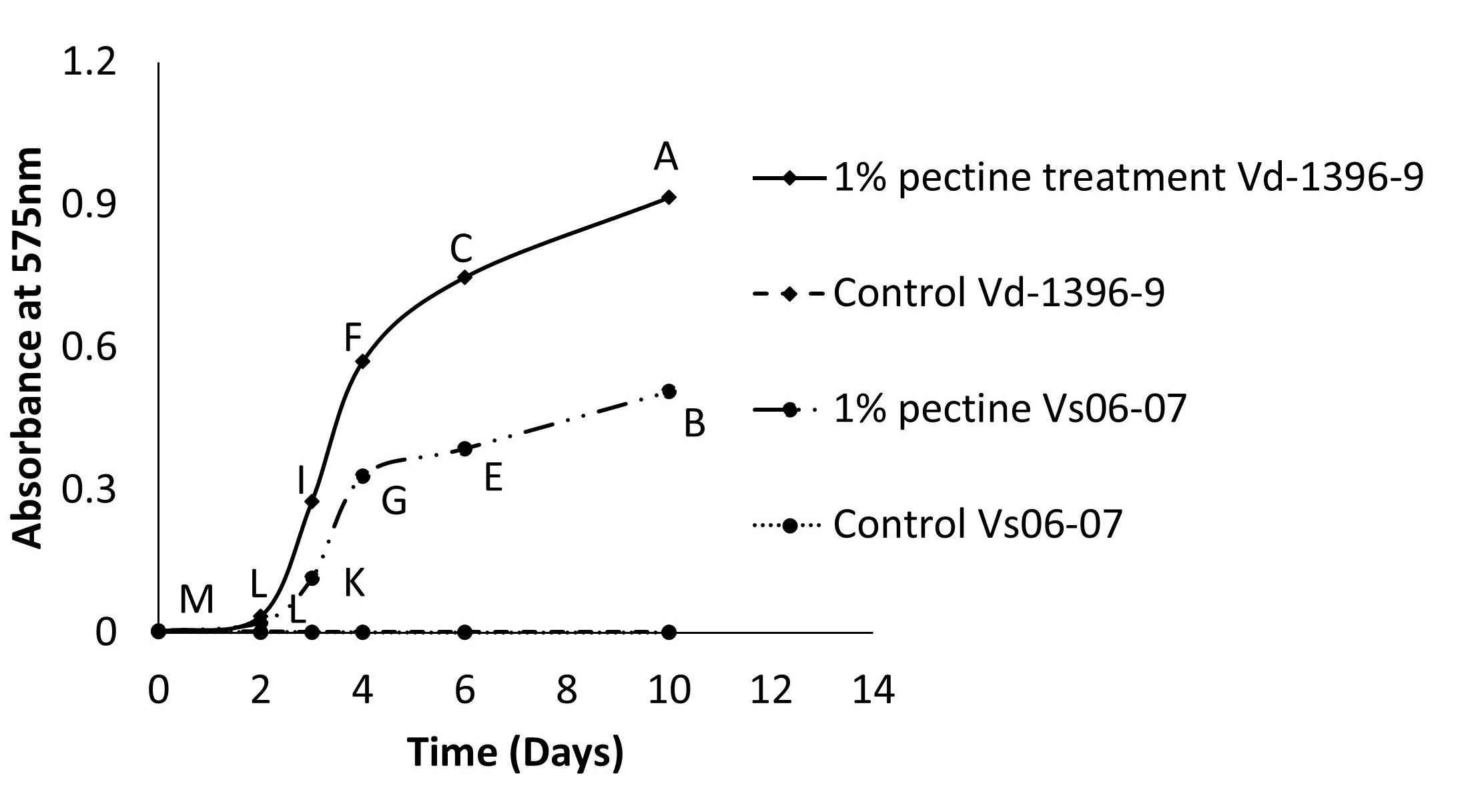

Supplement: Supplementary file 1 [file pathogens-10-00642-s001.zip › Supplementary figure S1.jpg]
